# Vitreoretinal Surgery in the Prevention and Treatment of Toxic Tumour Syndrome in Uveal Melanoma: A Systematic Review

**DOI:** 10.3390/ijms221810066

**Published:** 2021-09-17

**Authors:** Mario R. Romano, Fiammetta Catania, Filippo Confalonieri, Piero Zollet, Davide Allegrini, Jessica Sergenti, Francesco B. Lanza, Mariantonia Ferrara, Martina Angi

**Affiliations:** 1 Department of Biomedical Science, Humanitas University, Via Montalcini 4, Pieve Emanuele, 20090 Milan, Italy; mario.romano@hunimed.eu (M.R.R.); fiammetta.catania@humanitas.it (F.C.); filippo.confalonieri@humanitas.it (F.C.); piero.zollet@humanitas.it (P.Z.); 2 Department of Ophthalmology, Humanitas Castelli, Via Mazzini 11, 24128 Bergamo, Italy; davide.allegrini@humanitas.it (D.A.); mariantonia.ferrara@gmail.com (M.F.); 3 Ocular Oncology Service, Department of Surgical Oncology, Fondazione IRCCS Istituto Nazionale dei Tumori, Via Venezian 1, 20133 Milan, Italy; jessica.sergenti@istitutotumori.mi.it (J.S.); francesco.lanza@istitutotumori.mi.it (F.B.L.)

**Keywords:** choroidal melanoma, toxic tumour syndrome, proton beam therapy, endoresection, endodrainage

## Abstract

Toxic tumour syndrome (TTS) is a particularly aggressive form of secondary vasculopathy occurring after radiation therapy of uveal melanoma due to the persistence of the necrotic tumour mass inside the eye. The development of TTS confers a particularly unfavourable functional and anatomical ocular prognosis, ultimately requiring enucleation in most cases if untreated. Vitreoretinal (VR) surgery has been successfully applied for treatment and prevention of TTS using both resecting and non-resecting techniques. In this systematic review, we aim to define characteristics of uveal melanomas benefiting the most from secondary VR surgery and to outline the optimal type and timing of VR intervention in such cases. Analysis of the literature reveals that endoresection should be performed within 3 months after radiotherapy to tumours thicker than 7 mm and with a largest basal diameter between 8 mm and 15 mm with post-equatorial location, especially after proton beam treatment. Alternatively, endodrainage remains a valid therapeutic option in eyes with macula-off retinal detachment, tumour diameter larger than 15 mm or ciliary body involvement. VR surgery can be successful in the management of TTS following radiotherapy for uveal melanoma when timing and indication are appropriately evaluated.

## 1. Introduction

**Figure 1:**
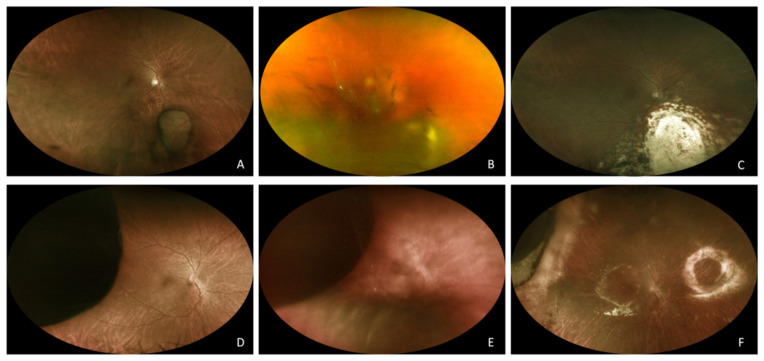
**Endoresection**. (**A**). Mushroom-shaped choroidal melanoma in the only eye with functional BCVA of an amblyopic patient at baseline. (**B**). Vitreous haemorrhage 6 months after PBT. (**C**). Surgical coloboma 3 years after PBT and 2.5 years after endoresection with final BCVA of 6/6. **Endodrainage**. (**D**). Large cilio-choroidal melanoma at baseline. (**E**). Macula off exudative retinal detachment 5 months after PBT. (**F)**. Tumour regression with attached retina under silicone oil 1 year after endodrainage.

Uveal melanoma can be successfully treated with conservative radiotherapy, such as brachytherapy, proton beam therapy (PBT), Gamma Knife radiosurgery (GKRS), or cyberknife (CK), providing targeted delivery of high radiation doses to the tumour while minimising collateral damage to the surrounding tissues [1]. Nevertheless, the inflammation induced by the persistence of necrotic irradiated tumour can give rise to a particularly aggressive form of secondary radiation vasculopathy [2,3]. Damato et al. [4] first introduced the term toxic tumour syndrome (TTS) referring to the combination of radiation retinopathy (RR), exudative retinal detachment (ERD), and neovascular glaucoma (NVG) occurring after radiotherapy for uveal melanoma. Tumour size, retinal and ciliary body invasion, diabetes mellitus, and retinal detachment at diagnosis are the most important predisposing factors, conferring a 5-year risk of 52% and 27% for NVG and ERD, respectively [5]. These elements enhance predisposition to retinal ischaemia and inflammation after radiotherapy. In fact, tumour necrotic cells cause activation of innate inflammatory response, leading to inflammatory cells infiltration and production of cytokines and vascular endothelial growth factor (VEGF). This process induces further increase in vascular permeability in radiation damaged tumour blood vessels, leading to massive oedema and transitory tumour mass enlargement. Presence of VEGF-producing viable retinal tissue, low oxygen tension, and reduced venous drainage are key elements for NGV development [6]. TTS confers an unfavourable ocular prognosis, ultimately requiring enucleation in most cases if untreated [5]. Excision of the dying tumour (endoresection) can prevent the development of TTS due to debulking of tumour necrotic tissue [1,7]. Even the sole removal of retinal exudate has been shown to reduce the incidence TTS, prompting the use of endodrainage as preventive strategy [8,9]. Clinical examples of endoresection (ER) and endodrainage are shown in Figure 1.

In this review of the literature we describe the role of vitreoretinal (VR) surgery in the prevention and management of TTS following radiotherapy for uveal melanoma [10]. The objective is to identify which tumours can benefit the most from secondary VR surgery and to define the optimal type and timing of VR intervention.

## 2. Methods

### 2.1. Search Methods

A comprehensive literature review was performed searching electronic databases, including the following: CENTRAL, Ovid MEDLINE, Embase, Clinical Trials.gov, and the World Health Organisation (WHO) International Clinical Trials Registry Platform (ICTRP). We did not use any date or language restriction in the electronic searches for trials. We last searched the electronic databases on 17 March 2021. The following keywords were used for the research: uveal melanoma, choroidal melanoma, choroidal melanoma endoresection, choroidal melanoma endodrainage, toxic tumour syndrome, neovascular glaucoma, local treatment failure, proton beam therapy, GKRS, CK, stereotactic radiotherapy, iodine-125 brachytherapy, and ruthenium brachytherapy.

Ethical review and approval were waived for this study, as it is a review of the literature. 

### 2.2. Selection Process

Types of study considered were clinical trials, case control studies, cohort studies, and case series including at least 5 patients.

We evaluated studies whose participants underwent secondary pars plana vitrectomy (PPV) with or without associated ER for treatment and prevention of TTS. 

**Figure 2:**
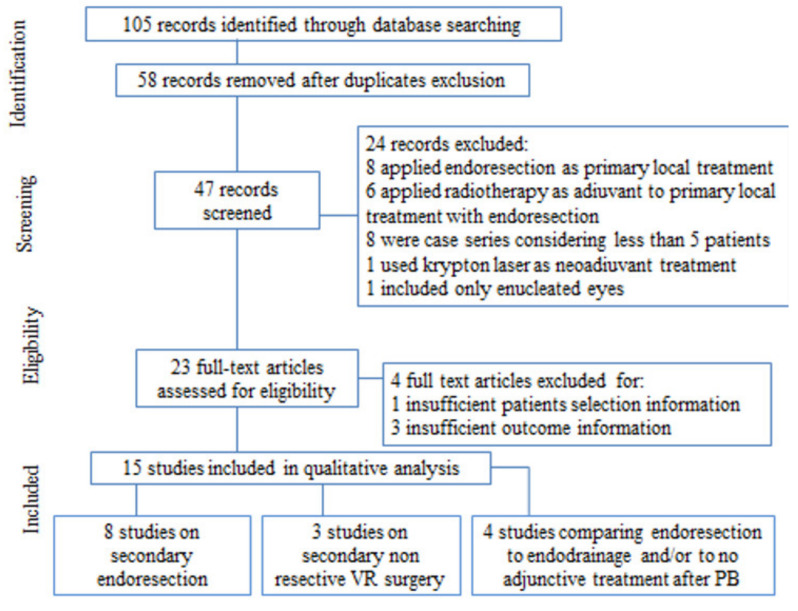
Visual representation of the selection process according to PRISMA guidelines.

Selected studies had to report type of radiation, surgical management, globe preservation rate, NVG rate with at least 1-year follow-up. Two authors independently screened search results, assessed trial quality, and extracted data using standard methodological procedures. A senior author supervised the process and solved any discrepancy. Our initial search yielded 105 reports, while the final selection resulted in the 15 articles included. Selection process and reasons for exclusion are detailed in a PRISMA diagram [11] (Figure 2).

## 3. Results and Discussion

### 3.1. Endoresection

Two prospective and one retrospective case series described the results of ER following PBT for a total of 213 patients (Table 1). Only 6 of them underwent ER for treatment of TTS, the indication for the rest being prevention of the condition in high-risk tumours. The 6 patients with established TTS presented with macula-off ERD located in the inferior quadrants and 2 patients manifested NVG. Globe preservation rate at the end of the study was 100% with no new case of NVG. Mean best corrected visual (BCVA) at baseline was 6/60. Only one patient retained a BCVA > CF after treatment. 

Patients undergoing ER for prevention showed tumour thickness ≥7 mm and posterior or equatorial location in all three studies. Kubicka-Trząska et al. [12] included only tumours with LBD <15.0 mm and Bechrakis et al. [13] excluded tumours with ciliary body and subfoveal infiltration and tumours with circumpapillary growth exceeding half of the optic disc circumference. The studies differed in time interval from radiotherapy to surgery (Table 1). Reasons for enucleation in the study from Bechrakis et al. [14] were phthisis bulbi (*n* = 1), complicated RD (*n* = 1) and NVG (*n* = 1). NVG occurred 18 months after surgery in a case of iuxtapapillary melanoma with LBD of 18.3 mm that was eventually enucleated. No case of NVG was detected in the other two studies. Despite a 100% globe preservation rate at last follow-up, Kubicka-Trząska et al. [12] reported two cases of phthisis bulbi. Probability of developing RR or radiation optic neuropathy after 24 months was estimated at 27.5% and 29.4%. Signs of RR or neuropathy were first seen at a median of 12 months after irradiation, with the earliest report at 6 months. Mean preoperatory BCVA significantly decreased during the study period in all analysed studies, with LBD as the only significant risk factor.

Three retrospective case series and one case control study [15,16,17,18] reported the outcome of ER after GKRS treatment (Table 1). Biewald et al. [15] performed adjuvant brachytherapy in most cases and included tumours with 19.3% of ciliary body involvement, 13.7% of optic nerve involvement, and a 95% presence of ERD at baseline. Both Schilling et al. [16] and Biewald et al. [15] excluded patients with extrascleral invasion and metastasis at baseline. Globe preservation rate ranged from 87% to 100% at 3 years follow-up. Biewald et al. [15] reported a mean time to enucleation of 12.55 months, indications for secondary enucleation were phthisis (59.5%), local recurrence (4%), severe vitreous haemorrhage (27.4%), and loss of fundus visibility (9.1%). Enucleation rate was significantly higher in tumours with ciliary body involvement, LBD > 15 mm, and greater thickness. Schilling et al. [16] noted 3 cases of secondary enucleation all due to NVG. Suesskind et al. [18] registered higher frequency of enucleation in eyes with ciliary body involvement, LBD > 18 mm and T4 size category. Suesskind et al. [18] reported a significantly lower rate of NVG, RR, and radiation optic neuropathy in ER + GKRS group compared to GKRS only group. In the latter, 4 cases of TTS and 12 cases of NVG developed during follow-up, compared to no cases in the ER-treated group. In the study from Sinyavskiy et al. [17], NVG and severe RR occurred in 19% of patients, all with long-standing ERD and delayed surgery. Schilling et al. [16] reported RR in only 1 patient and noted that recurrence of ERD was associated with tumour volume. Patients receiving additional adjuvant brachytherapy [15] did not have a significant difference in final BCVA or eye retention rate. In the study from Suesskind et al. [18], median BCVA loss was inversely proportional to distance to the optic disc.

Lastly, McCannel et al. [19] reported the results of ER following iodine-125 brachytherapy. Three out of 5 patients showed signs of RR before ER. At the end of the follow-up, no enucleation and only 1 case of NVG were described. All 5 patients displayed RR in the form of macular oedema and peripheral ischaemia by the end of the study.

**Table 1:** Summary of studies on secondary endoresection following different types of radiotherapy. CF = counting fingers; ERD = exudative retinal detachment; GKRS = gamma knife radiosurgery; HM = hand movement; LBD = largest basal diameter; NVG = neovascular glaucoma; PBT = proton beam therapy; RT = radiotherapy; BCVA = best corrected visual acuity.

Author(s), Reference	Number of Analysed Eyes	Inclusion Criteria	Type of RT	Time from RT to Surgery	LBD (mm)	Tumour Thickness (mm)	Follow-Up Duration (Months)	Globe Preservation Rate	NVG Rate	ERD Recurrence Rate	Preop BCVA	Final BCVA
Bechrakis NE et al., *Int Ophthalmol Clin*. 2006 [14]	58	Postequatorial tumours ≥7 mm thickness	PBT	9 days (4–21)	15.6 (11.1–21.6)	8.8 (7–14.5)	18 (3–48)	91.6%	1.7%	32.1%	20/40 (20/20–20/400)	20/200 (20/50-HM)
Kubicka-Trząska A, *Arch Med Sci-Civiliz Dis*. 2020 [12]	13	Postequatorial tumours LBD ≤ 15.0 mm, thickness ≥ 8.0 mm	PBT	5.6 weeks (3–9)	13.4 (10.6–15.0)	7.6 (5.8–9.3)	61.6 (34–84)	100%	0%	23.1%	6/50 (6/7.5–CF)	30.1% ≥ 6/15 69.9% CF-NLP
Bechrakis NE et al., *Klin Monbl Augenheilkd*. 2009 [13]	142	Posterior tumours ≥ 7 mm thickness	PBT	12 days (3–20)	N/A	N/A	30	97%	0%	N/A	N/A	N/A
Sinyavskiy OA, J *Neurosurg*. 2016 [17]	21	Equatorial or posterior LBD ≥ 10 mm	GKRS	20 days (3–97)	13.9 (10.0–18.2)	8.9 (31.-15.5)	23 (8–41)	100%	19%	14.3%	≥20/50: 33.3% 20/50–20/400: 28.6% ≤20/400: 38.1%	≥20/50: 23.8% 20/50–20/400: 42.8% ≤20/400: 33.3%
Biewald E, Br J *Ophthalmol*. 2017 [15]	200	≥ 8 mm thickness Absence of NVG	GKRS	10 days	12 (6.3–20)	9.4 (6–14.8)	32.3	89%	0%	30%	≥20/50: 49.5% 20/50–20/400: 37.1% ≤20/400: 13.4%	≥20/50: 13.4% 20/50–20/400: 33.6% ≤20/400: 53%
Schilling, Klin *Monbl Augenheilkd*. 2006 [16]	43	LBD < 15 mm >7 mm thickness >3 mm distance from foveola or optic disc	GRKS		11.4 ± 2.1	9.5 ± 1.4	13.7	87%	7%		20/50	20/80
Suesskind, *JAMA Ophthalmol*. 2013 [18]	18	>6 mm thickness, touching the optic disc	GKRS		14.22 ± 3.28	8.31± 1.50	23.0 (0.1–81.1)	87%	0%		20/50 (20/20–20/200)	median loss of Snellen lines–22 (−38 to 0)
McCannel TA, *Eye (Lond)*. 2013 [19]	5	Mild signs of radiation retinopathy	I-125-brachytherapy	26.8 months (13–62)	NA	5.8 (2.03–8.9)	62.4 (30–117)	100%	20%	0%	20/120 (20/400–20/25)	CF (NLP-20/70)

### 3.2. Endodrainage and Other Non-Resecting Techniques

Three studies focused on the results of non-resecting VR techniques in the prevention and treatment of TTS (Table 2). Seibel et al. retrospectively described outcomes of endodrainage in the prevention of TTS following PBT. Indications for endodrainage were large ERD at diagnosis in patients with severe comorbidities, ciliary body involvement, and LBD >18 mm. PPV and oil tamponade were successful in reattaching the retina in 100%. Enucleation was performed in 1 patient (5.0%) 45 months after PPV because of NVG. In total, secondary glaucoma was detected in 5 cases (20.0%) at average 9.3 months from PBT. RR occurred in 40.0% and radiation optic neuropathy in 35.0% of patients with a mean interval of 7.1 and 9.2 months, respectively. Visual acuity continued to drop during follow-up and remained stable only 3 years after PBT.

Two studies described the results of PPV and silicone oil tamponade during I-125 brachytherapy. This practice is based on the evidence of an attenuating effect of silicone oil 1000-cSt, resulting in a reduction of 50–60% of radiation exposure of healthy ocular tissue to side radiation from iodine-125 brachytherapy [20]. Lyons et al. [21] reported 2 cases of RR and 1 case of NVG that was eventually enucleated. BCVA was particularly well preserved in the cohort. McCannel et al. [22] reported a globe preservation rate of 95% in patients undergoing Iodine-125 brachytherapy alone compared to 100% rate and significantly higher BCVA in patients treated with external drainage of exudative retinal detachment, PPV and silicone oil tamponade. 

### 3.3. Endoresection Compared to Endodrainage and to No Adjuvant Surgical Treatment after PBT

Seibel et al. published 2 case control studies comparing ER and endodrainage after PBT in the prevention of TTS (Table 3). One of the two studies also included a control group treated with PBT alone. The study including all the 3 groups [24] analysed 903 patients, counting 50 enucleations by the end of follow-up. Kaplan–Meier analysis showed an overall eye retention rate of 94.8% after 5 years and 93.2% after 10 years. Enucleation rate was significantly higher in control (9.7%) than in the ER (4.3%) or endodrainage (4.1%) group. Additional risk factors for enucleation were involvement of ciliary body or optic disc. NVG represented the cause for enucleation in 95.3% of the cases in control group, 70% of the cases in endodrainage group and 36.8% of the cases in ER group. Moreover, enucleation due to NVG occurred at a median time of 27 months, 33 months, and 38 months in the control, endodrainage and ER groups, respectively. Other reasons for enucleation were local recurrence, which was significantly more frequent in ER group, and scleral necrosis (10% of enucleations in both surgical groups). RR was diagnosed at a mean of 18 months in all the 3 groups, with controls showing higher 5-year rates (52.3%) compared with endodrainage (37.4%) or ER (30.5%) groups. NVG occurred in 11.6% after 28.3 months in the ER group, which was significantly lower than 21.3% (after a mean of 26.7 months) and 57.8% (after a mean of 24.3 months) in the endodrainage and control groups, respectively. Seibel et al. [25] documented in both groups 90% retinal reattachment rate with significant visual impairment during follow-up, even though endodrainage produced a slightly lower BCVA loss than ER. Furthermore, ER was associated with higher incidence of RR and radiation optic neuropathy, possibly being confounding factors due to the posterior location of tumours suitable for ER.

Schönfeld et al. [26] published a retrospective report of equatorial choroidal melanoma following PBT, 71% of whom underwent ER as prevention for TTS. Criteria for ER were poorly defined, with indications including large sized tumours with thickness ≥ 6 mm. Enucleation was performed in 1 case per group. Indications for late enucleation were phthisis bulbi and local recurrence. NVG rate was 23% for PBT alone and 18% for PBT associated with ER. BCVA loss was more relevant in patients treated with secondary ER.

Cassoux et al. [27] assessed the efficacy of ER compared to no adjunctive treatment and transpupillary thermotherapy (TTT) in PBT patients. Most patients presented with LBD between 10 and 15 mm (59%), thickness between 5 and 10 mm (81%) and ERD at diagnosis (79.4%). Ciliary body invasion and optic disc involvement were present in 8% each. NVG was significantly less frequent in the ER group compared to the others, showing an incidence of 7% in 2 years compared to 49% in TTT group and 58% in PBT only group. Secondary enucleation was necessary for NVG in 24.5% of patients in the PBT only group and in 9.8% of patients in the TTT group. Two cases of phthisis were reported when ER was performed for tumours with LBD > 16 mm. There was no significant difference in BCVA loss after treatment between groups.

### 3.4. Discussion

First line therapies for radiation vasculopathy and TTS are anti-VEGF and steroidal intravitreal medications combined with retinal laser photocoagulation [12]. Among second line therapies, TTT has shown positive results in both prevention and treatment of TTS [7]. These treatments, however, are not always effective, particularly when the radiation dose is high and the necrotising tumor is large. Therefore, most authors performed VR surgery after radiation treatment for tumours located post-equatorially, with thickness ≥ 7 mm and LBD ≥ 8 mm. In fact, tumour height, LBD, volume, and posterior location are the most significant predictive factors for TTS development. In particular, centrally located tumour with a large distance to equator and close distance (<2.5 mm) to sensitive structures bear the highest probability to develop the condition [29]. A possible explanation for the relationship with tumour dimensions is the exponential production of VEGF from retinal pigment epithelium cells and choroidal fibroblast cells in response to higher local levels of IFN-γ, TNF-α and IL-1β [30], which are proportional to necrotising tumour mass’ size. Higher basal levels of cytokines, VEGF, and VEGF-R could also explain the increased rate of TTS in diabetic patients. Nevertheless, the analysed studies did not consider diabetes. Although the susceptibility to radiation vasculopathy reported in posterior tumours is partially explained by the direct irradiation and damage of the more densely vascularised posterior structures, the pathogenetic mechanism subtended to higher rates of TTS in posterior tumours is less clear. We postulate posterior pole to be more prone to VEGF production in response to inflammatory conditions. Consistently, some authors detected retinal areas with higher density of photoreceptors to be more prone to vascular survival and homeostatic function of constitutive VEGF and to the presence of a more abundant VEGF reservoir [31]. As a confirmation, Schönfeld S et al. [26] analysing only equatorially located tumours, found little difference in terms of enucleation and NVG rate despite great differences in tumour dimensions between the group undergoing ER and controls. Optic disc and ciliary body involvement represent risk factors for TTS and enucleation after secondary VR surgery in some of the analysed studies.

A few studies [14,25] considered patients with manifest TTS and the role of VR surgery in the resolution of the condition. In such instances, globe preservation rate and recurrence of ERD did not vary significantly between prevention and treatment groups. At the end of follow-up, patients manifesting TTS at baseline showed a universally poor BCVA while patients treated for prevention of TTS retained a good BCVA in some cases. Sinyavskiy et al. [17] suggested that, independently from tumour dimensions, long-standing large macula-on or small to large macula-off ERD are an absolute indication for ER. In fact, tumour exudation is a risk factor for permanent visual loss due to apoptotic and necrotic cell death mechanisms leading to significant loss of photoreceptors [23]. Despite the fact that non-surgical management with intravitreal bevacizumab leads to 73% resolution of ERD [32], this outcome is obtained at a mean of 4 months from the injection, sufficient time to cause irreversible BCVA deterioration [9]. The need for prompt surgical treatment of ERD is further confirmed by the risk of developing subretinal fibrosis associated with longstanding detachment [14]. Another potential indication is the presence of hemophthalmos, which is also indicative of massive necrosis and breakdown of the internal blood-retinal barrier [33].

By contrast, ER must be carefully performed in tumours with LBD larger than 15 mm due to an augmented risk of local surgical complications [12]. The most dangerous intraoperative complication related to ER is air embolism through vortex veins [34,35], which can occur for tumours of any size. In the study from Suesskind et al. [18], ER after GKRS was suspended due to 3 cases of unexplained sudden death immediately after surgery. In the same study, more surgery-related enucleations (i.e., expulsive haemorrhage, persistent hypotonia, or painful phthisis bulbi) were registered in tumours with ciliary body involvement. Other complications of all described surgical techniques are inadvertent retinal touch at the macula, lens touch, inability to flatten detached retina, subretinal, and subchoroidal oil. The major postoperative complications include bleeding at the scleral bed (100%), cataract (about 50%), retinal detachment (about 30%), ocular hypertension (25%–30%), CNV (8%), epiretinal proliferation (10%), macular traction produced by chorioretinal scarring at the limits of the resected area, branch vein occlusion, and submacular haemorrhage. 

The most effective treatment for prevention of NVG and secondary radiation maculopathy is ER. Regarding globe preservation rate and recurrence of ERD though, endodrainage and ER show similar outcome while endodrainage seems to result in a better BCVA preservation. As a result, endodrainage is to be considered a valid preventive strategy in cases of ineligibility for ER due to non-replaceable anticoagulants, severe hypertension, ciliary body or macular involvement, or LBD exceeding 18 mm. In addition, PPV with silicone oil tamponade is an effective method to prevent NVG and enucleation in large posterior tumours treated with iodine-125 brachytherapy. In all VR surgical techniques, an interval comprised between 1 week and 3 months from radiotherapy to surgery seems to represent the best timing for prevention of TTS.

Limitations of this review are the small number of patients in some of the analysed studies, the retrospective nature of most of them and the time span between studies. Indeed, the more recent introduction of anti-VEGF treatment protocols for prevention of NVG may have influenced results as compared to older studies where such treatment was not available. Prospective multicenter studies are needed to further investigate the optimal use of medical and surgical treatment in the prevention of TTS.

## 4. Conclusions

Treatment of radiation-induced ocular complications has become an important part of uveal melanoma patients’ management. Tumors located post equatorially, with thickness ≥ 7 mm and LBD ≥ 8 mm and associated exudative retinal detachment are at risk of developing TTS and may need secondary enucleation. This can be prevented by performing VR surgery in the first trimester following radiotherapy.

## Figures and Tables

**Table 2:** Summary of case series on non-resective vitreoretinal surgery techniques in the prevention of toxic tumour syndrome following different types of radiotherapy. LBD = largest basal diameter; NVG = neovascular glaucoma; PBT = proton beam therapy; PPV = pars plana vitrectomy; SO = silicon oil; BCVA = best corrected visual acuity.

Author(s), Reference	Number of Analysed Eyes	Type of Radiotherapy	Time from Radiotherapy to Surgery (Months)	LBD (mm)	Tumour Thickness (mm)	Follow-Up Duration (Months)	Globe Preservation Rate	NVG Rate	Preop BCVA (logMAR)	Final BCVA (logMAR)
Seibel I et al., *Ocul Oncol Pathol*. 2014 [23]	20	PBT	4.5 (0.1–9.2)	14.2 (7.7–18.5)	6.2 (3.8–9.9)	38.4 (12.0–122.0)	95%	5%	1.1 (2.0–0.5)	2 years post: 1.4 (2.0–0.4) 3 years post: 1.7 (2.2–0.7) 4 years post: 1.8 (2.2–1.0)
McCannel TA, *Retina* 2017 [22]	20 PPV + SO + I-brachytherapy	I-125-brachytherapy		16.4 (1.7)	7.8 (2.7)	19.4 (12.2–36.7)	100%	5%	0.16 (0.21)	0.83 (0.86)
	20 I-brachytherapy			17.5 (1.8)	7.9 (2.1)	22.5 (12.0–56.2)	95%	0%	0.54 (0.59)	2.06 (1.4)
Lyons LJ, *Cureus.* 2019 [21]	5	I-125-brachytherapy		12.1 (9.7–14.5)	6.2 (3.18–11.02)	45 (12–56)	80%	20%	0.32	0.28

**Table 3:** Summary of selection criteria and outcome data from comparative studies on endoresection and endodrainage applied for the prevention of toxic tumour syndrome after proton beam treatment. EDR = endodrainage; ER = endoresection; PBT = proton beam; RD = retinal detachment; LBD = largest basal diameter; TTT = transpupillary thermotherapy; BCVA = best corrected visual acuity.

	Seibel I et al., *Am J Ophthalmol*. 2017 [24]			Cassoux N, *Retina* 2013 [27]			Seibel I et al., *Invest.* *Ophthalmol. Vis. Sci.* 2013 [25]	Schönfeld S et al., *Am J Ophthalmol*. 2014 [26]		
	PBT +ER	PBT + EDR	PBT	PBT + ER	PBT	PBT + TTT	PBT + ER	PBT + EDR	PBT + ER	PBT
Number of eyes	445	242	216	63	57	51	183	28	44	18
Inclusion criteria	≥7 mm thickness ≥8 mm LBD Exudative RD			>6 mm thickness >10 mm LBD			≥7 mm thickness ≥8 mm LBD Exudative RD	≥6 mm Large size		
Follow-up duration (months)	53.9 (12–156.5)	34.8 (12–102.7)	60.6 (29–169.3)	23 (20–26)	112 (107–126)	99 (89–124)	45.4 (2.8–123.5)	27.3 (4.2–95.1)	64.4	77.2
Tumour thickness (mm)	10.0 (6.0–16.4)	7.8 (5.9–11.2)	8.1 (7–15.1)	9 (6–12.2)	8 (5.1–11.3)	8 (5.3–11.8)		6.49± 2.17 (1.91–10.2)	9.1± 1.5	3.9 ± 2.0
LBD (mm)	14.5 (8.0–21.5)	14.7 (12.1–26.1)	14.4 (8.0–21.9)	14 (8–19)	17.5 (10–23.3)	17.4 (12.5–22.7)			13.7 ± 3.2	10.6 ± 3.0
Retinal detachment	100%	100%	100%	74.9%			100%	100%		
Distance to fovea	2.7 (0–12.1)	2.4 (0–19.0)	2.1 (0–21.5)						5.1 ± 2.2	3.7 ± 1.9
Distance to optic disc	2.3 (0–13.2)	2.4 (0–17.0)	3.1 (0–18.7)						5.3 ± 2.3	3.9 ± 1.9
T [28]	T1:1.6% T2: 19.7% T3: 56.6% T4: 22.0%	T1:3.3% T2: 16.5% T3: 42.6% T4: 37.6%	T1: 0 T2: 35.6% T3: 44.0% T4: 20.4%	T1 0% T2 19% T3 75% T4 6%	T1 0% T2 2% T3 51% T4 47%	T1 0% T2 0% T3 63% T4 37%				
AJCC prognostic staging [28]	I: 1.6% IIA:19.5% IIB: 51.5% IIIA: 20.7% IIIB: 6.3% IIIC: 0.4%	I: 2.1% IIA: 25.2% IIB: 33.5% IIIA: 26.0% IIIB: 11.1% IIIC: 2.1%	I: 0% IIA: 4.2% IIB: 28.7% IIIA:46.3% IIIB:18.0% IIIC: 2.8%							
Enucleation rate	19 (4.3%)	10 (4.1%)	21 (9.7%)	3 (6%)	14 (25%)	5 (10%)			1 (2.3%)	1(5.5%)
NGV rate	54 (12.1%)	51 (21.1%)	126 (58.3%)	4 (7%)	33 (58%)	25 (49%)			4 (18.6%)	4 (23.5%)
Preoperatory BCVA				<1/100: 17% 1/100 to 20/50: 59% >20/50: 24%	<1/100: 18% 1/100 to 20/50: 55% >20/50: 27%	<1/100: 10% 1/100 to 20/50: 70% >20/50: 20%	0.35 logMAR (20/45)	0.5 logMAR (20/60)	20/16–20/50 = 59.1% 20/63–20/160 = 31.8% <20/200 = 9.1%	20/16–20/50 = 83.3% 20/63–20/160 = 11.1% <20/200 = 5.6%
Final BCVA				<1/100: 68% 1/100 to 20/50: 13% >20/50: 5%	<1/100: 67% 1/100 to 20/50: 28% >20/50: 5%	<1/100: 72% 1/100 to 20/50: 26% >20/50: 2%	1.0 logMAR (20/200)	0.8 logMAR (20/120)	20/16–20/50 = 18.2% 20/63–20/160 = 20.4% <20/200 = 61.4%	20/16–20/50 = 50.0% 20/63–20/160 = 16.7% <20/200 = 33.3%
